# Fisetin Prevents Angiogenesis in Diabetic Retinopathy by Downregulating VEGF

**DOI:** 10.1155/2023/7951928

**Published:** 2023-02-03

**Authors:** Meihua Lai, Caifeng Lan, Junmu Zhong, Lijuan Wu, Chengmin Lin

**Affiliations:** ^1^Department of Ophthalmology, Longyan First Affiliated Hospital of Fujian Medical University, Longyan, Fujian 364000, China; ^2^Department of Ultrasonography, Longyan First Affiliated Hospital of Fujian Medical University, Longyan, Fujian 364000, China; ^3^Department of Ophthalmology, Wenzhou Hospital of Integrated Traditional Chinese and Western Medicine, Wenzhou, Zhejiang 325000, China

## Abstract

Diabetic retinopathy (DR) is one of the more serious complications of diabetes. However, the mechanisms involved in DR are complex and still need to be investigated. The beneficial effects of fisetin have been widely reported, but whether it is beneficial in DR is not clear yet. This study was designed to investigate the regulatory role of fisetin in regulating DR and explore the involved mechanism. First, 30 mM glucose was used to establish DR cell model *in vitro.* Cell counting kit 8 (CCK8) assay was utilized to study the effects of fisetin on cell viability through treating human retinal microvascular endothelial cells (HRMECs) with 25, 50, and 100 *μ*M fisetin. Transwell and wound healing assays were used to detect the function of fisetin on the migration and angiogenesis on HG-induced HRMECs. Finally, OE-VEGF was used as a mimic of VEGF, and western blotting (WB) was used to verify the targeting genes of fisetin. HG induced an increase in cell viability, cell migration, and angiogenesis in HRMECs, whereas fisetin inhibited these enhancements induced by HG through inhibiting VEGF. In conclusion, fisetin prevents angiogenesis in DR by downregulating VEGF.

## 1. Introduction

Among the complications of diabetes, diabetic retinopathy (DR) is the most serious. As a vascular complication, this complication has a complex mechanism and is often caused by multiple factors [1–5]. Relevant references suggest that the retina of diabetic patients responds rapidly to hyperglycemia, leading to an imbalance between pro- and anti-angiogenic processes. Hyperglycemia can trigger a range of deficiencies in biological functions, including not only damage to retinal capillaries, but also abnormal vasoconstriction. The mechanisms involved may be related to increased secretion of angiogenic factors.

Angiogenic factors have been shown to play a regulatory role in the pathogenesis of retinal neovascularization (NV), including vascular endothelial growth factor (VEGF), the most important one. Studies have shown that its expression can be detected in many cells, not only in retinal endothelial cells, but also in Müller cells, retinal pigment epithelium (RPE) cells, etc. In addition, astrocytes and ganglion cells also express this growth factor. However, overexpression of VEGF results can lead to excessive formation of new blood vessels, which in turn results in vessel leakage. It is reported that the migration, proliferation, and tubular formation of human retinal endothelial cells (HRECs) are caused by VEGF through autocrine secretion during the disease process. In addition, VEGF can also regulate angiogenesis in other cells, mainly through paracrine secretion. It is evident that VEGF is a potential therapeutic target for angiogenesis during DR [6]. Therefore, anti-VEGF drugs are considered as an ideal medication for the treatment of DR. Uemura et al. summarized that VEGF is important for retinal angiogenesis [7]. Michael concluded that targeting VEGF is beneficial for DR patients [8].

Fisetin is a flavonoid polyphenol molecule that is widely found in various fruits and vegetables such as strawberries, apples, onions, and cucumbers, with the highest content in strawberries. Several pharmacological benefits of fisetin have been reported, including anti-inflammatory, anti-apoptotic, antioxidant, antitumor, and anti-angiogenic effects [9]. For instance, Maher found that the neuroprotective and anti-inflammatory effects of fisetin were associated with the transition of metal ions [10]. It inhibits HG-induced vascular inflammation [11]. Chen et al. found that fisetin could protect cells from apoptosis by activating the IGF-IR-PI3K-Akt signaling pathway [12]. Althunibat et al. found that fisetin alleviated diabetic cardiomyopathy by ameliorating hyperglycemia-induced inflammation, oxidative stress, and apoptosis [13]. Several studies have collectively concluded that fisetin decreases the progression of cancers via suppressing signaling pathways, such as NF-*κ*B and PAK4 [14, 15]. Besides, fisetin may exert its function by regulating cytokine production and inhibiting NF-*κ*B activation in the retina [16]. It also inhibits angiogenesis by inhibiting the VEGF/VEGFR signaling pathway and can be used as a candidate drug for inhibiting angiogenesis in retinoblastoma [17]. Current studies have not found any side effects of fisetin, but several reports claim that it may cause stomach upset and interfere with medications. In addition, it has been suggested that the low solubility and low bioavailability of fisetin limit its investigation [18]. Furthermore, this study aimed to investigate the role if fisetin in DR and potential mechanism.

## 2. Methods

### 2.1. Cells and Treatment

Human retinal microvascular endothelial cells (HRMECs) were obtained from Li's lab (Capital Medical University, Beijing, China) and cultured in M199 medium (Solarbio, Beijing, China), which is prepared by mixing with 10% fetal bovine serum (FBS; Solarbio, Beijing, China). When the medium was used, 1% penicillin and streptomycin (Corning, Somerville, Massachusetts, USA) were added. HRMECs were cultured in conditioned medium of 5 mM or 30 mM d-glucose at 37°C and 5% CO_2_. The experimental grouping scheme was as follows: control group, NG; HG group, HG; HG plus 25 *μ*M fisetin group, HG + 25 *μ*M fisetin; HG plus 50 *μ*M fisetin group, HG + 50 *μ*M fisetin; and HG plus 100 *μ*M fisetin group, HG + 100 *μ*M fisetin.

### 2.2. CCK8

CCK8 kit (Lablead, Beijing, China) was used to detect the viability of HRMECs treated with d-glucose for 48 h. The absorbance of cells at 450 nm was measured separately using a microplate reader (SpectraMax i3X, Molecular Devices, CA, USA). The experiment was repeated for three times.

### 2.3. Transwell Assay

After treatment of d-glucose on HRMECs for 48 h, single-cell suspensions were prepared and resuspended in serum-free DMEM. Transwell assays were performed in a BD Matrigel™ Invasion Chamber (MA, USA). Low concentration of serum with DMEM was used to prepare cell suspension (50,000), which was then added to the upper chamber and the lower chamber filled with 0.7 mL DMEM supplemented with 10% FBS. Cells were incubated at 37°C for 24 h in a humidified incubator containing 5% CO_2_. After incubation, cells under the membrane were treated with 800 *μ*L crystal violet (Cat No. C8470; Solarbio, Beijing, China) at room temperature and stained for 30 minutes and then counted in 3 random high-power fields using a light microscope. For invasion experiments, chambers precoated with matrix adhesive (Corning Inc., NY, USA) were applied and all experiments were repeated for 3 times.

### 2.4. Wound Healing Assay

A 24-well culture plate was used to culture HRMECs at approximately 10,000 cells per well. The culture conditions were as described above, with a duration of 24 hours. Then, the culture medium was aspirated. To create cell wound surface, 10 μL pipette tips were used. Afterwards, cells were washed using sterile PBS and next added 1mL of medium. The cell growth and migratory distance at the scratch site were observed and recorded at 0 hours and 24 hours. The experiment was repeated for three times.

### 2.5. Tube Formation Assay

To precool a 96-well plate, add 60 *μ*L of Matrigel (BD Biosciences, San Jose, CA, USA) to each well. The HRMEC suspension was then homogeneously seeded on the hardened Matrigel and incubated in an incubator for 24 hours. Afterwards, an inverted microscope was used to observe cell morphology and changes. Three fields of view were randomly selected to observe tube formation in each well, and Image J software was used to count and quantify capillary branch points around cells.

### 2.6. Immunofluorescence Staining

Cells were fixed with 4% paraformaldehyde and incubated with anti-VEGF (Cat no. 19003-1-AP; Proteintech, Shanghai, China) overnight at 4°C. Then, HRP Anti-Rabbit IgG antibody (Cat no. ab288151; Abcam, Cambridge, United Kingdom) was used. Nuclei were blocked with DAPI for 10 min. Coverslips were observed by an inverted fluorescence microscope (Axiocam 702 mono, Zeiss, Germany).

### 2.7. Western Blot

To obtain total protein from cells, high-efficiency RIPA lysate (Cat no. R0010; Solarbio, Beijing, China) was used. Then, the speed of the high-speed centrifuge was set at 16,000 × *g*, the temperature was set at 4°C, and the centrifugation was performed for a total of 15 min. The protein concentration was then quantified by BCA protein assay kit (Cat no. PC0020, Solarbio, Beijing, China). The protein was denatured after being treated at 95°C for 5 min, followed by SDS-PAGE (7.5%), and the loading amount of protein per pore was 30 *μ*g. After electrophoresis, the protein was transferred to PVDF membrane (Solarbio, Beijing, China) by wet transfer method, blocked with 5% skimmed milk dissolved in TBST prepared in advance, and placed on a shaker for 2 h at room temperature. The corresponding primary antibodies were used to incubate the membranes.

The primary antibodies were as follows: VEGF (Cat no. 19003-1-AP; Proteintech, Shanghai, China) and GAPDH (1 : 2,000) (Cat no. 60004-1-Ig; Proteintech, Shanghai, China). Then, the membranes were incubated with the horseradish peroxidase (HRP)-conjugated secondary antibody (1 : 2000) (Solarbio, Beijing, China) for 2 h at 37°C. ECL Western Blotting Substrate (Solarbio, Beijing, China) was used to detect the intensity of chemiluminescence. Image J version 1.53 was used to analyze results.

### 2.8. Statistical Analysis

The results of all experiments are presented as mean ± standard deviation (SD), and the number of experiments was repeated for 3 times. When comparing differences between two groups, the *t*-test was used, and when comparing differences among more than 3 groups, one-way analysis of variance (ANOVA) was used. Data were tested for normality, and data that do not conform to normality were tested by Wilcoxon rank sum test. GraphPad Prism version 6.0 software (GraphPad Software, Inc., La Jolla, CA, USA) was applied. *P* < 0.05 indicated a significant difference.

## 3. Results

### 3.1. Fisetin Inhibits HG-Induced Cell Viability in HRMECs

**Figure 1:**
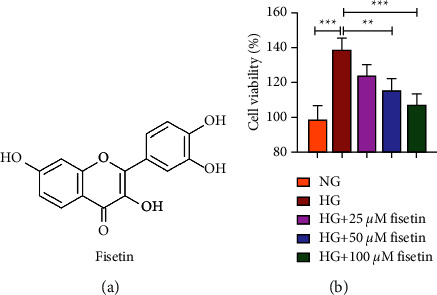
Chemical structure of fisetin and its effect on the viability of HRMECs. (a) Chemical structure of fisetin. (b) Effects of 25, 50, and 100 *μ*M fisetin on the viability of HRMECs after treated with 5 and 30 mM d-glucose for 48 h.

The chemical structure of fisetin is shown in Figure 1(a), which indicated that fisetin carries four hydroxyl groups and has good hydrophilicity. The effect of fisetin on cell viability after treatment of HRMECs with 5 mM or 30 mM d-glucose was detected by CCK8. It was found that HG significantly increased the viability of HRMECs relative to NG, while both HG + 50 *μ*M and HG + 100 *μ*M fisetin significantly reduced the HG-induced cell viability (Figure 1(b)). It can be concluded that fisetin decreases cell viability in a dose-dependent manner.

### 3.2. Fisetin Inhibits HG-Induced Cell Migration in HRMECs

**Figure 2:**
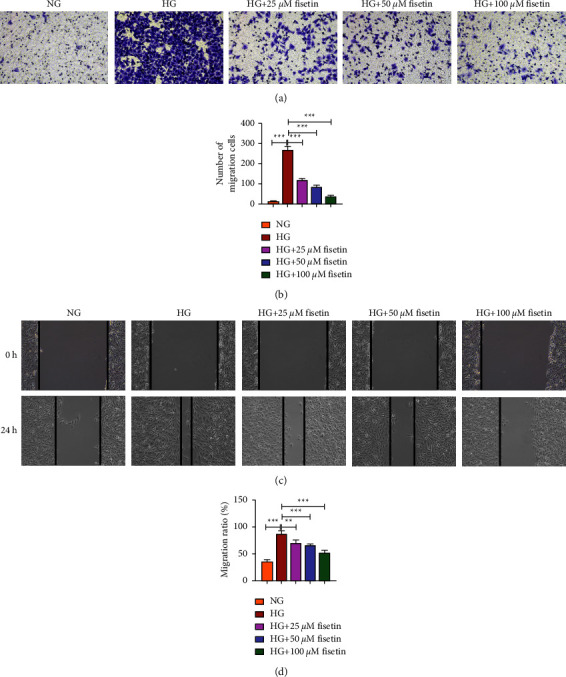
The effect of fisetin on HG-induced migration of HRMECs. (a) Transwell assay was used to detect the migration of HRMECs. (b) Statistics of (a) under different fields of view. (c) The migratory width of HRMECs within 24 h in the scratch assay. (d) Statistics of (c) under different fields of view.

The effect of fisetin on cell migration induced by HGHG was further investigated using Transwell and wound healing assays (Figures 2(a) and 2(b)). The amount of purple crystals was closely related to cell progression. Results from Transwell assay showed that under the microscope, the NG group had a sparse number of crystals, whereas the HG group significantly increased the number of purple crystals in the field of view. Compared to the HG group, fisetin at 25 *μ*M, 50 *μ*M, and 100 *μ*M all significantly reduced the HG-induced crystals. The wound healing assay showed consistent results. Compared to NG group, HG significantly increased the migratory width of cells within 24 hours. Compared to HG group, fisetin at 25 *μ*M, 50 *μ*M, and 100 *μ*M significantly reduced the HG-induced cell migration (Figures 2(c) and 2(d)). Taken together, fisetin could suppress cell migration, invasion, and progression in a dose-dependent manner.

### 3.3. Fisetin Inhibits HG-Induced Angiogenesis in HRMECs

**Figure 3:**
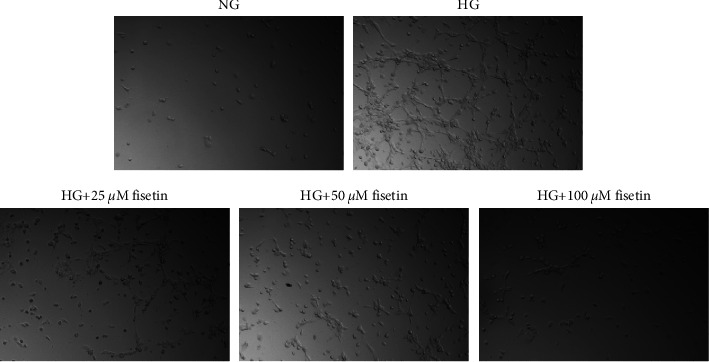
Fisetin induces tube formation in HRMECs induced by HG.

Previous experiments found that fisetin inhibited HG-induced cell migration, generating interest in its effects on angiogenesis (Figure 3). No significant angiogenesis was investigated in the NG group, whereas angiogenesis was significantly induced in the HG group. Relative to the HG group, fisetin at 25 *μ*M, 50 *μ*M, and 100 *μ*M showed a dose-dependent effect on HG-induced angiogenesis.

### 3.4. Fisetin Inhibits VEGF Expression in HRMECs

**Figure 4:**
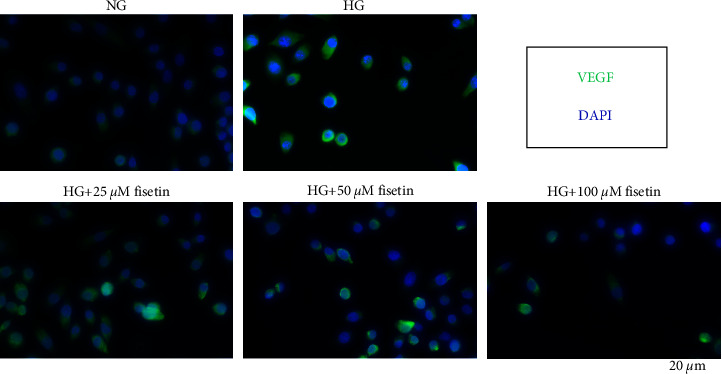
The effect of fisetin on the expression of VEGF in HRMECs induced by HG. Blue represents DAPI (nucleus) and green represents VEGF.

Immunofluorescence was used to detect the expression of VEGF under co-treatment of HG and different concentrations of fisetin. The results showed that in the NG group, there was no significant fluorescence of VEGF. Compared to the NG group, the green fluorescence of VEGF in the HG group was significantly increased, indicating that the expression of VEGF was activated, whereas the VEGF expression of fisetin at 25 *μ*M, 50 *μ*M, and 100 *μ*M gradually decreased as compared to HG treatment (Figure 4). It was suggested that fisetin could inactivate VEGF expression in cells in a dose-dependent manner.

### 3.5. Fisetin Attenuates HG-Induced Migration and Angiogenesis by Inhibiting VEGF in HRMECs

**Figure 5:**
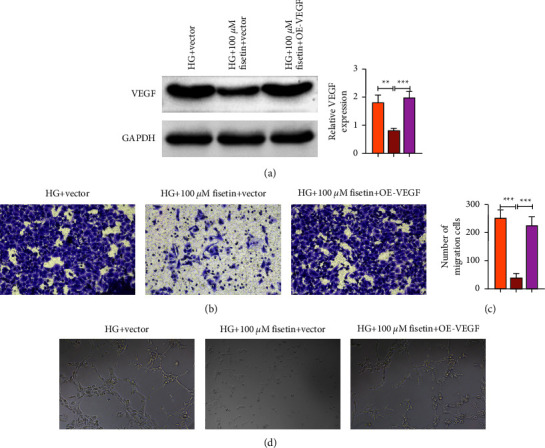
Effects of fisetin with (without) VEGF on the expression of VEGF and cell behavior in HRMECs induced by HG. (a) The effect of fisetin with (without) VEGF treatment on the expression of VEGF. (b) Transwell assay was used to detect the effect of fisetin with (without) VEGF treatment on the migration of HRMECs. (c) Effects of fisetin with (without) VEGF treatment on cell tube formation of HRMECs.

To further investigate how fisetin affects the behavior of HRMECs, the VEGF analogue OE-VEGF was used to investigate its interaction with fisetin. VEGF is a gene that is closely related to the cell progression and growth. Western blotting results showed that 100 *μ*M fisetin significantly reduced the HG-induced VEGF expression compared to the HG + vector group, whereas the addition of OE-VEGF reversed the trend of this inhibition (Figure 5(a)). Furthermore, the results of Transwell assay showed that there was substantial cell migration in the HG + vector group, whereas 100 *μ*M fisetin significantly reduced HG-induced cell migration, but the addition of OE-VEGF increased cell migration (Figure 5(b)). Similarly, substantial angiogenesis could be found in the HG + vector group, but it was inhibited by 100 *μ*M fisetin, and the addition of OE-VEGF promoted angiogenesis (Figure 5(c)). Taken together, it can be concluded that fisetin inactivates the progression of migration and angiogenesis in a dose-dependent manner.

## 4. Discussion

In this study, the viability of HRMECs was induced by HG. It has been reported that HG can induce a significant increase in the viability of retinal endothelial cells [19]. This is consistent with our results. However, this increase in cell viability can cause a range of functional impairments that further aggravate and exacerbate visual impairment.

Cell migration is an important part of the formation of multicellular tissues and also plays an important role in wound healing. In our experiments, no significant increase in cell migration was found in the NC group, while HG induced an increase in the migration of HRMECs. Furthermore, fisetin inhibits the migratory ability of HG-induced HRMECs in a dose-dependent manner. This observation was consistent with previous studies. Yan et al. found that HG induced cell migration, and protective drugs reduced this migration [20]. Wang et al. also found that HG induced cell migration, and these studies suggest that cell migration is critical in the progression of diabetes [21]. The decrease in cell migration after fisetin treatment suggests that disease progression can be inhibited. These studies also found that angiogenesis was also mostly induced by HG, and our experimental results were consistent with the findings of angiogenesis. Long et al. found that breviscapine, also a natural chemical component, prevents angiogenesis in DR by downregulating VEGF/ERK/FAK/Src pathway signaling [6]. However, the inhibition of angiogenesis by fisetin used in this study was dose-dependent, indicating that fisetin may also inhibit angiogenesis in DR. However, which genes are regulated by fisetin in DR needs to be investigated.

VEGF plays a central role in mediating microvascular and macrovascular pathology in diabetes [22]. VEGF is a major mediator of DR and is capable of inducing the changes observed in proliferative retinopathy, macular edema, and possibly nonproliferative DR. Aiello and Wong found that fisetin inhibits VEGF-induced angiogenesis in retinoblastoma cells, and the specific mechanism is inhibition of cell migration and angiogenesis [22]. Coincidentally, Farooqi et al. in his systematic review summarized the anticancer effects of fisetin, including the regulation of VEGF/VEGFR signaling [23]. These studies support our conclusion that fisetin can attenuate HG-induced migration and angiogenesis by inhibiting VEGF.

However, this study has flaws. First, this study did not establish animal models to validate the results. In the future, animal models would be set to verify the conclusion from this study. Second, although fisetin has shown its beneficial pharmacological effects, its safety still needs to be evaluated, such as whether it affects other normal cells. After all, the composition of retinal cells is not monolithic but complex. Third, fisetin inhibited angiogenesis in DR through inactivating the expression of VEGF, but other possible effects have not been determined. In addition, the research on how to apply or transfer current concentration in clinical practice has not been investigated in this study; therefore, a series of experimental design should be further investigated. However, this study still provides some new insights for clinical drug practice.

## 5. Conclusion

In conclusion, the present study found that fisetin prevents angiogenesis in DN by downregulating VEGF.

## Data Availability

All data generated or analyzed during this study are included within the article.
